# ﻿Review of the millipede genus *Malayorthomorpha* Mršić, 1996 (Diplopoda, Polydesmida, Paradoxosomatidae), with descriptions of two new species from Thailand and a key to its species

**DOI:** 10.3897/zookeys.1118.89593

**Published:** 2022-08-16

**Authors:** Natdanai Likhitrakarn, Sergei I. Golovatch, Wisut Sittichaya

**Affiliations:** 1 Program of Agriculture, Faculty of Agricultural Production, Maejo University, Chiang Mai, 50290, Thailand; 2 Biodiversity and Utilization Research Center of Maejo University, Maejo University, Chiang Mai, 50290, Thailand; 3 Institute for Problems of Ecology and Evolution, Russian Academy of Sciences, Leninsky pr.33, Moscow 119071, Russia; 4 Agricultural Innovation and Management Division, Faculty of Natural Resources, Prince of Songkla University, Hat Yai, Songkhla, 90112, Thailand

**Keywords:** Malaysia, *Malayorthomorphahalabala* sp. nov., *Malayorthomorphahulutbeeda* sp. nov., Orthomorphini, taxonomy

## Abstract

The millipede genus *Malayorthomorpha* Mršić, 1996, so far monospecific and previously known only from Park Belum, Perak State, northern Malaysia, is recorded from a mountain in Betong District, Yala Province, southern Thailand for the first time, being represented there by two new species: *M.halabala***sp. nov.** and *M.hulutbeeda***sp. nov.** Both new species are found to occur syntopically and can be assumed as narrowly endemic to the Titiwangsa Mountain Range which begins in southern Thailand, crosses the Malaysian border, and extends into east and west coast regions of the Malay Peninsula. In addition, the generic diagnosis is slightly updated, and a key to all three species is provided.

## ﻿Introduction

The millipede genus *Malayorthomorpha* Mršić, 1996 was established for a single, and type species, *Malayorthomorphasiveci* Mršić, 1996, based on two males from northern Malaysia (Mršić 1996). This genus was immediately assigned, and still belongs, to the tribe Orthomorphini Brölemann, 1916, all 25 genera of which are basically confined to the Oriental Region (Nguyen and Sierwald 2013; Srisonchai et al. 2018a, b, c, d). The tribe is characterized by the gonopod that shows an elongate (not shortened) femorite and both solenophore (= tibiotarsus) and solenomere of medium size, the former’s both mesal and lateral lobes (a lamina medialis and a lamina lateralis, respectively) sheathing, supporting and protecting a flagelliform solenomere (Jeekel 1968). The latest key to the genera of the tribe belongs to Golovatch (1997), but presently it is considerably out of date.

Thailand is located in the central part of mainland Southeast Asia within two significant biodiversity hotspots, Indo-Burma and Sundaland (Myers et al. 2000), both outstanding in supporting an especially rich and diverse diplopod fauna (Likhitrakarn et al. 2011, 2020; Pimvichai et al. 2018, 2020). To date, the Thai millipede list comprises 256 species in 52 genera, 17 families and nine orders, largely reported based on explorations during 2007–2022 throughout Thailand (e.g., Pimvichai et al. 2018, 2020; Srisonchai et al. 2018a, b, c, d, 2021; Likhitrakarn et al. 2020, 2021a, b, 2022; Rosenmejer et al. 2021; Bhansali and Wesener 2022).

However, there are still some areas that have never been explored and prospected sufficiently well for millipedes, such as three southern border provinces within the Malay Peninsula: Pattani, Narathiwat and Yala. Some Diplopoda have only been documented from the Yala and Narathiwat provinces, while the Pattani Province has remained devoid of any millipede records (Enghoff 2005). Only ten species have been reported from the Yala Province (Table 1), nine of which share a single locality, the Bang Lang National Park (5°30'7"N, 101°26'21"E). Four species are only known from one locality, and three from two localities with a range of less than 50 km^2^. These seven species are presumably endemic to the country or restricted to a small area in the Malay Peninsula. However, only relatively small areas have been prospected, with just five sampling locations in the three provinces that have provided reports of millipede species.

**Table 1.** Localities of millipede species recorded from the Yala and Narathiwat provinces, Thailand.

No.	Species	Locality	Remark
**Order Sphaerotheriida**			
1	*Sphaerobelummeridionalis* Bhansali & Wesener, 2022	Yala Province, Than To District, Bang Lang National Park (Than To Waterfall), 150 m a.s.l., 6°11'47.5"N, 101°09'50.9"E (Bhansali and Wesener 2022).	
**Order Spirostreptida**			
2	*Anurostreptusbarthelemyae* Demange, 1961	Yala Province, Bang Lang National Park, 6°04'N, 101°11'E; Narathiwat Province, Khao Mala (Enghoff 2005).	Originally described from Peninsular Malaysia (Demange 1961) and also reported from the Satun and the Songkhla Province, Thailand (Enghoff 2005).
3	*Thyropygusaterrimus* (Pocock, 1889)	Yala Province, Bang Lang National Park, 6°11'47.5"N, 101°09'50.9"E; Naratiwat Province, Waeng District, Hala-Bala WS Research Station, 5°47'44.8"N, 101°50'4.2"E (Enghoff 2005).	Also known from Myanmar (Pocock 1889) and Malaysia (Sinclair 1901; Wang and Tang 1965).
4	*Thyropygusfloweri* (Demange, 1961)	Bukit Jalor (=Yala) (Demange, 1961); Yala Province, Bang Lang National Park, 6°04'12"N, 101°11'18"E (Pimvichai et al. 2009).	
**Order Polydesmida**			
5	*Eutrichodesmuscavernicola* (Sinclair, 1901)	Yala Province, Mueang Yala District, Wat Khuhapimuk (adjust the precise position of Sinclair (1901) based on Huber et al. (2015) by Srisonchai et al. (2020)).	
6	*Anoplodesmusmalayanus* (Golovatch, 1993) (E)	Records from Thailand: Yala Province, Bang Lang National Park, 6°04'N, 101°11'E, <400m (Golovatch 1993).	
7	*Desmoxytesdelfae* (Jeekel, 1964)	Yala Province, Bang Lang National Park, lowland rainforest, 6°4'N, 101°11'E (Srisonchai et al. 2018a).	The species was found in abundance in the provinces of Surat Thani, Krabi, Nakhon Si Thammarat, Phatthalung, Trang, Satun, and Songkhla, which cover the majority of southern Thailand (Srisonchai et al. 2018a).
8	*Haplogonomorphagogalai* Mršić, 1996	Yala Province, Bang Lang National Park, 6°04'N, 101°11'E, <400m (Golovatch 1998).	This monotypic species was originally described from Peninsular Malaysia (Mršić 1996).
9	*Orthomorphabanglangensis* Golovatch 1998	Yala Province, Bang Lang National Park, 6°04'N, 101°11'E (Golovatch 1998).	
10	*Substrongylosomamoniliforme* Golovatch, 1993	Yala Province, 20 km south of Tham To, 5°50'N, 101°10'E, 200 m; Yala Province, Bang Lang National Park, 6°04'N, 101°11'E, 400 m a.s.l. (Golovatch 1993).	

Luckily, we have recently been privileged to survey an evergreen forest in the Betong District, Yala Province near the Thai-Malaysia border during the rainy season. Based on morphological examinations of the new specimens, we are able to herewith describe and illustrate two new species of the genus *Malayorthomorpha* which is formally reported from Thailand for the first time.

## ﻿Materials and methods

New material was collected in a montane forest at a rather high elevation near the Thai-Malaysian border. The specimens collected were euthanized by a two-step method following the AVMA Guidelines for the Euthanasia of Animals (AVMA 2013). Material was then preserved in 75% ethanol for morphological observations and brought to the laboratory. The specimens were examined, measured and photographed under a Nikon SMZ 745T trinocular stereo microscope equipped with a Canon EOS 5DS R digital SLR camera. Digital images obtained were processed and edited with Adobe Photoshop CS5. Line drawings were based on photographs and examined under the stereo microscope equipped with a digital SLR camera. For scanning electron microscopy (SEM), the gonopods were coated with 8 nm gold layer using a CCU-010 high vacuum sputter and a carbon coater (Safematic), then imaged with a TESCAN VEGA3 scanning electron microscope operated at 5 keV of acceleration voltage and returned to alcohol after SEM examination. The holotypes and paratype are housed in the Museum of Zoology, Chulalongkorn University (**CUMZ**), Bangkok, Thailand.

In the synonymy sections, **D** stands for the original description and/or subsequent descriptive notes, **K** for the appearance in a key, **L** for the appearance in a species list, while **M** for a mere mention.

The terminology concerning gonopodal and somatic structures mostly follows Mršić (1996), Golovatch and Enghoff (1993), Golovatch (1997), and Srisonchai et al. (2018b, 2018c). Abbreviations of certain gonopodal structures are as follows:

**g** groove, a distinct groove line running parallel to the solenomere, clearly seen in mesal view

**ll** lamina lateralis, a flat lobe in the distal part of the gonopod

**lm** lamina medialis, a large part located distally on the gonopod, tapered apically and unciform

**sl** solenomere, usually a long and flagelliform structure originating at the base of the solenophore

**sph** solenophore (= tibiotarsus), the apical part of the telopodite, consisting of a lamina lateralis and a lamina medialis

The Animal Care and Use Protocol Review No. 1723018 was applied.

Coordinates and elevations were recorded by Garmin GPSMAP 60 CSx and Garmin eTrex 30 using the WGS84 datum and subsequently double-checked with Google Earth ver. 7.3.4

## ﻿Taxonomy

### ﻿Family Paradoxosomatidae Daday, 1889

**Subfamily Paradoxosomatinae Daday, 1889**

#### Tribe Orthomorphini Brölemann, 1916

##### ﻿Genus Malayorthomorpha

Taxon classificationAnimaliaPolydesmidaParadoxosomatidae

Mršić, 1996

CD7AEBAE-D2F2-54E9-B704-2AB9C3B52C9A

Malayorthomorpha Mršić, 1996: 139 (D).
Malayorthomorpha – Golovatch 1997: 134 (M, K); Shelley et al. 2000: 111 (L).

###### Amended diagnosis.

Body medium-sized to large (ca. 24–41 mm long, ca. 1.2–2.7 mm wide), with 20 segments. Paraterga from poorly to rather well developed, without lateral incisions. Transverse metatergal sulcus distinct. Leg relatively long and slender, without modifications. ♂ tarsal brushes absent. Sternal lobe between ♂ coxae 4 present, other sternites unmodified.

Gonopods rather simple to relatively complex; coxites elongate, subcylindrical, sparsely setose distoventrally, without tubercles; prefemoral (= setose) part of telopodite moderate to relatively large, 1/3–1/2 as long as acropodite; femorite moderately long and stout, slightly curved, devoid of a distinct distolateral sulcus demarcating a postfemoral part; a well-developed lamina medialis and a hypertrophied lamina lateralis of solenophore; the latter subterminally with a long, distally pointed and curved lobe broadened at base and protecting the tip of a curved solenomere. Apex of solenophore subquadrate. Solenomere flagelliform, starting about level to demarcation cingulum between femorite and solenophore, seminal groove running entirely or mostly mesally along an excavate femorite.

###### Type species.

*Malayorthomorphasiveci* Mršić, 1996, by original designation.

###### Affinities.

As noted earlier (Golovatch 1997, 1998), the gonopodal conformation of *Malayorthomorpha* seems to especially similar to that of *Cleptomorpha* Golovatch, 1997, a monospecific genus of Orthomorphini from Sumatra, Indonesia. Yet both genera compared differ clearly in the gonopod femorite showing an indistinct, oblique, mesal fold, a relatively slender solenophore and an apically terminating solenomere in *Cleptomorpha* compared to the gonopod femorite that is clearly excavated mesally, has a considerably stouter solenophore, and the solenomere termnating mesally about the solenophore midway in *Malayorthomorpha* (Golovatch 1997).

### ﻿Key to species of *Malayorthomorpha*, chiefly based on ♂ characters

**Table:** 

1	Sternal lobe between ♂ coxae 4 linguiform with a rounded tip (Figs 6H, I, 7E). Gonopod lamina lateralis (**ll**) triangular in shape, bifid at tip, and protruded laterally (Figs 7B–D, 8A, C–F)	***Malayorthomorphahulutbeeda* sp. nov.**
–	Sternal lobe between ♂ coxae 4 deeply notched medially (Figs 1D, 3H, I, 4E). Gonopod lamina lateralis (**ll**) elevated and expanded apically (Figs 1E–G, 4A–D, 5)	**2**
2	Pleurosternal carinae present until segment 11. Sternal lobe between ♂ coxae 4 with a pair of small cones near base (Figs 3H, I, 4E). Gonopod tip with a denticulate margin (Figs 4A, B, 5C, D)	***Malayorthomorphahalabala* sp. nov.**
–	Pleurosternal carinae present until segment 5. Sternal lobe between ♂ coxae 4 without cones near base (Fig. 1D). Gonopod tip with a smooth margin (Fig. 1E–G)	***Malayorthomorphasiveci***

#### ﻿ Malayorthomorpha siveci

Taxon classificationAnimaliaPolydesmidaParadoxosomatidae

Mršić, 1996

7C98A142-7D1B-537D-A34B-9149772328D3

Fig. 1

Malayorthomorpha siveci Mršić, 1996: 139 (D).
Malayorthomorpha siveci – Shelley et al. 2000: 111 (L).

##### Remark.

This species was described from Park Belum, 5°30'7"N, 101°26'21"E, ca. 320–350 m a.s.l., Hulu (Sungani), Perak, Malaysia (Mršić 1996). Only two male specimens have been obtained, and both have been discovered in a small area. This species is considered endemic to northern Malaysia.

**Figure 1.**
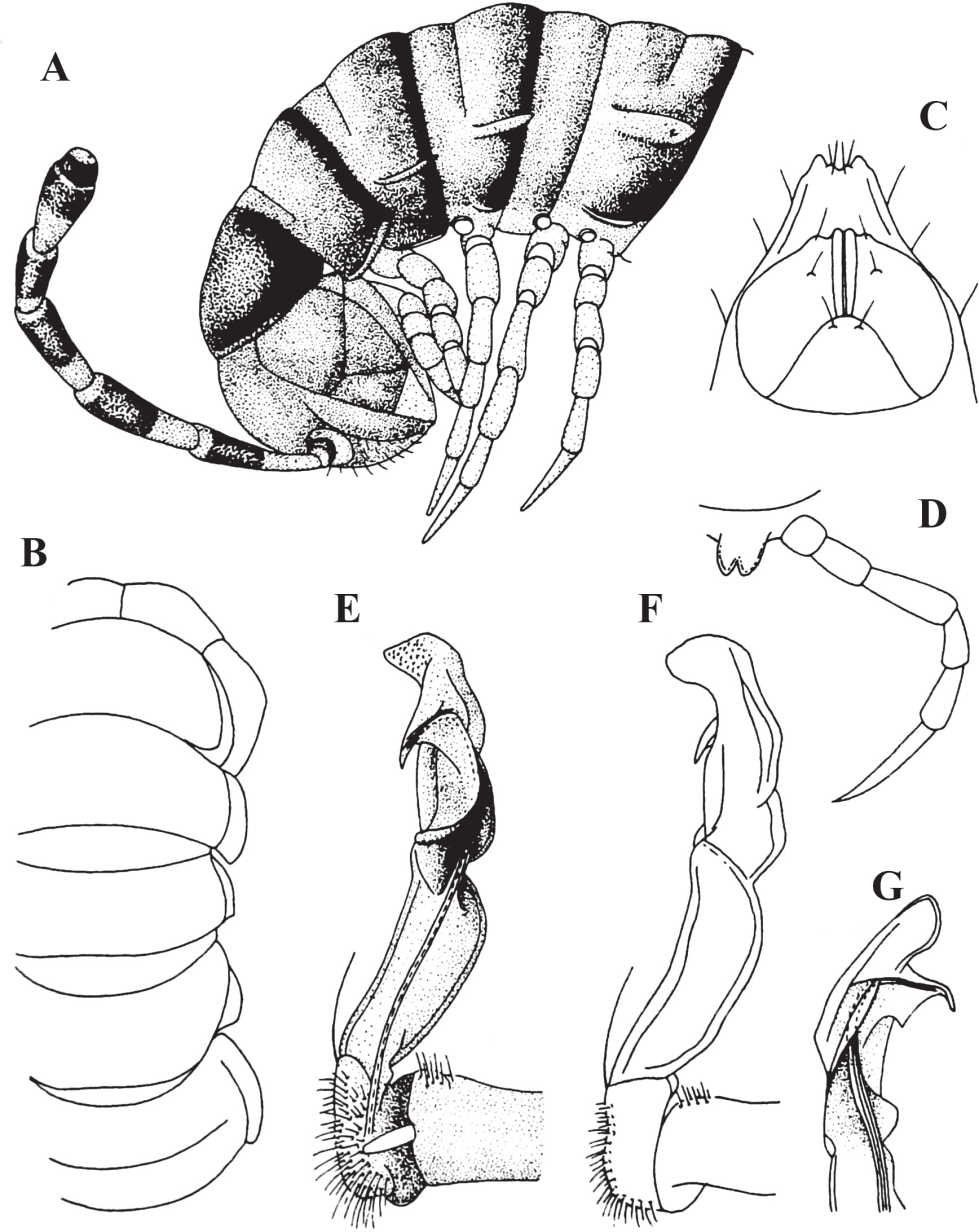
*Malayorthomorphasiveci* Mršić, 1996, ♂ holotype **A, B** anterior part of body, lateral and dorsal views, respectively **C** anal segment, ventral view **D** sternal process and left anterior leg of body segment 5, suboral view **E–G** right gonopod, mesal, lateral and suboral views, respectively. Photos not to scale (after Mršić 1996).

#### ﻿ Malayorthomorpha halabala sp. nov.

Taxon classificationAnimaliaPolydesmidaParadoxosomatidae

13904BA5-24F8-5CCB-A8D4-BE005104B34C

https://zoobank.org/3FB913EC-E071-4A1A-A306-9657A857AFDB

Figs 2A , 3 , 4 , 5

##### Material examined.

***Holotype***: Thailand – Yala Province • ♂; Betong District, hill in evergreen forest, on forest floor; 1440 m a.s.l.; 5°55'N, 101°26'E; 22 May 2021; Wisut Sittichaya leg.; CUMZ. ***Paratype***: Thailand – Yala Province • ♀; same District, elfin montane forest (Malaya Phytochorion province); 1430 m a.s.l.; 25 May 2022; Wisut Sittichaya leg.; CUMZ.

##### Diagnosis.

This new species seems to be particularly similar to *M.siveci* Mršić, 1996, with which it shares most of the gonopodal characters. It differs from *M.siveci* by the wider body, 2.7–3.2 mm (vs smaller, 1.2 mm), the colour pattern which is uniformly red brown with lighter red brown paraterga (Fig. 3A–F) (vs a light brown body with the collum and caudal edges of metazonae margined darker brown; Fig. 1A), as well as the pleurosternal carinae present until segment 11 (vs until segment 5), the sternal lobe between ♂ coxae 4 with a pair of small cones laterally near base (Fig. 3E, H, I) (vs absent, Fig. 1D), and the tip of the gonopod with a denticulate margin (Figs 4A, B, 5C, D) (vs smooth and rounded; Fig. 1E–G).

##### Description.

Length 29.3 (♂) or 36.2 mm (♀), width of midbody pro- and metazonae 2.1 and 2.7 mm (♂) or 2.7 and 3.2 mm (♀), respectively.

**Figure 2.**
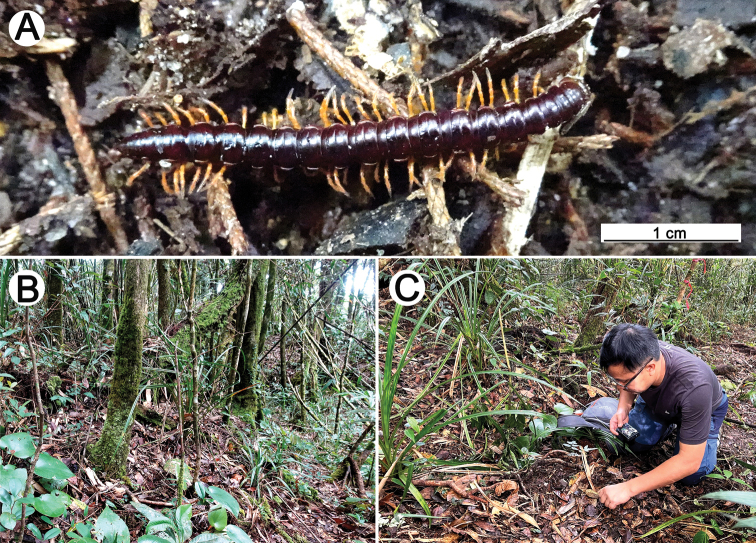
Habitat at the type locality of *Malayorthomorphahalabala* sp. nov., ♀ **A** live colouration **B, C** elfin montane forest floor and collecting the specimens **B, C** pictures taken not to scale.

**Figure 3.**
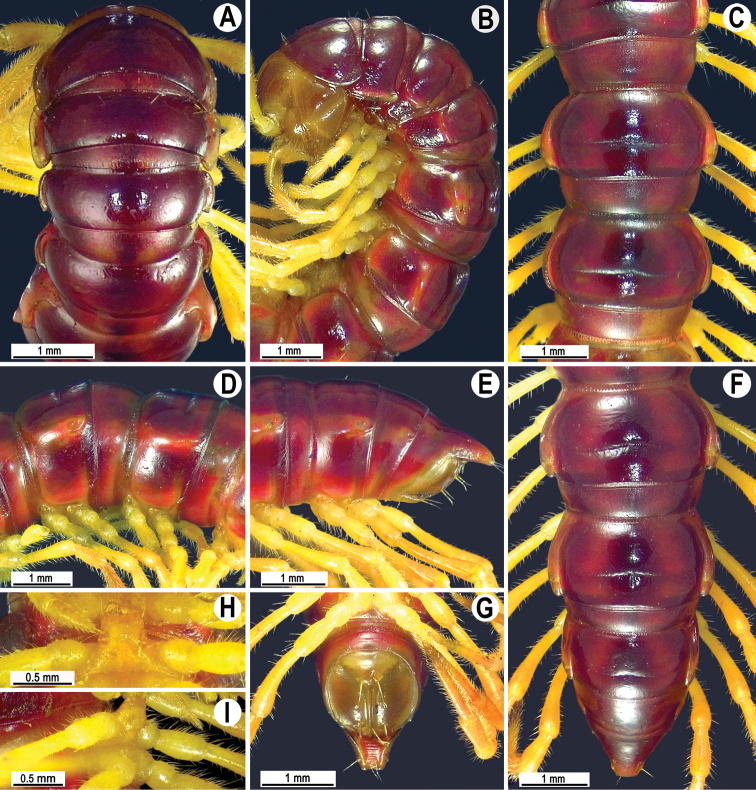
*Malayorthomorphahalabala* sp. nov., ♂ holotype **A, B** anterior part of body, dorsal and lateral views, respectively **C, D** segments 10 and 11, dorsal and lateral views, respectively **E–G** posterior part of body, lateral, dorsal and subventral views, respectively **H, I** sternal cones between coxae 4, subcaudal and sublateral views, respectively.

Colouration of live animal rusty red (Fig. 2A), edges of paraterga light red brown; antennae dark brownish, legs and venter contrasting light yellow (Fig. 2A); colouration in alcohol, after one week of preservation, red brown (Fig. 3A–F); edges of paraterga light red brown, head and antennae brown, legs, venter and a few basal antennomeres contrasting light yellow (Fig. 3A–G).

Clypeolabral region sparsely setose; epicranial suture distinct. Antennae long, extending caudally past metaterga 5 (♂) or metaterga 3 (♀) when stretched dorsally. In width, segment 3 < 4 = collum < segment 2 = head < segment 5 < 6–17, body gently and gradually tapering thereafter.

Collum with three transverse rows of setae: 4+4 in anterior, 2+2 in intermediate, and 3+3 in posterior row, all mostly abraded, but still traceable as insertion points; lateral incisions absent; caudal corner of paraterga very broadly rounded, declined ventrad, produced slightly past rear tergal margin (Fig. 3A, B).

Tegument generally smooth and shining, prozonae finely shagreened, metaterga finely leathery and faintly rugulose (Fig. 3A, C, F), surface below paraterga leathery and rugose (Fig. 3B, D, E). Postcollum metaterga with two transverse rows of setae traceable at least as insertion points when setae broken off: 2+2 in anterior (presulcus) and 3+3 in posterior (post-sulcus) row. Tergal setae simple, slender, ca. 1/3 as long as metaterga. Axial line barely traceable both on pro- and metazonae.

Paraterga rather well developed (Fig. 3A, C, F), lying rather high (at upper 1/3 of body), slightly upturned, but lying below dorsum; anterior edge broadly rounded and narrowly bordered, fused to callus; lateral edge without incisions; caudal corner very narrowly rounded, not produced past rear tergal margin except in rings 2 and 3 (Fig. 3A, B); posterior edge nearly straight. Paraterga 2 broad, anterior edge angular and rounded, lateral edge without incisions (Fig. 3A).

Calluses on paraterga rather narrow, delimited by a sulcus fully on dorsal side and in about posterior 2/3 on ventral side; on poreless rings more narrow than on pore-bearing ones in dorsal view (Fig. 3B, D, E). Ozopores evident, lateral, lying in an ovoid groove at about 1/3 in front of posterior edge of metaterga.

Transverse sulcus usually distinct (Fig. 3A, C, F), complete on metaterga 5–17, narrow, line-shaped, rather deep, not reaching the bases of paraterga, very faintly ribbed at bottom, incomplete and nearly wanting on segment 18. Stricture between pro- and metazona wide, deep, ribbed at bottom down to base of paraterga starting with segment 5 (Fig. 3A–E, F). Pleurosternal carinae complete crests with a sharp caudal tooth on rings 2–4, increasingly reduced and retaining a sharp caudal tooth on rings 5 and 6 thereafter, further retained as a small caudal tooth and increasingly reduced until segment 11, absent from segment 12 on (♂, ♀) (Fig. 3B, D, E).

Epiproct (Fig. 3E–G) conical, flattened dorsoventrally, with two evident, but small, rounded, apical papillae; tip subtruncate; pre-apical papillae small, but evident, lying close to tip. Paraprocts regularly convex, each with premarginal sulci medially and two pairs of setigerous knobs at medial margin (Fig. 3G). Hypoproct roundly subtrapeziform, setigerous knobs at caudal edge very small and well-separated (Fig. 3G).

Sterna sparsely setose, shining, cross-impressions shallow, without modifications; a single, linguiform, medially rather deeply notched sternal lobe between ♂ coxae 4, with a pair of small cones laterally near base (Fig. 3E, H, I). A conspicuous and high ridge present in front of gonopod aperture. Legs long and slender (Fig. 3B), midbody ones ca. 1.4–1.6 (♂) or 1.2–1.3 (♀) times as long as body height, without modifications, ♂ tarsal brushes absent.

**Figure 4.**
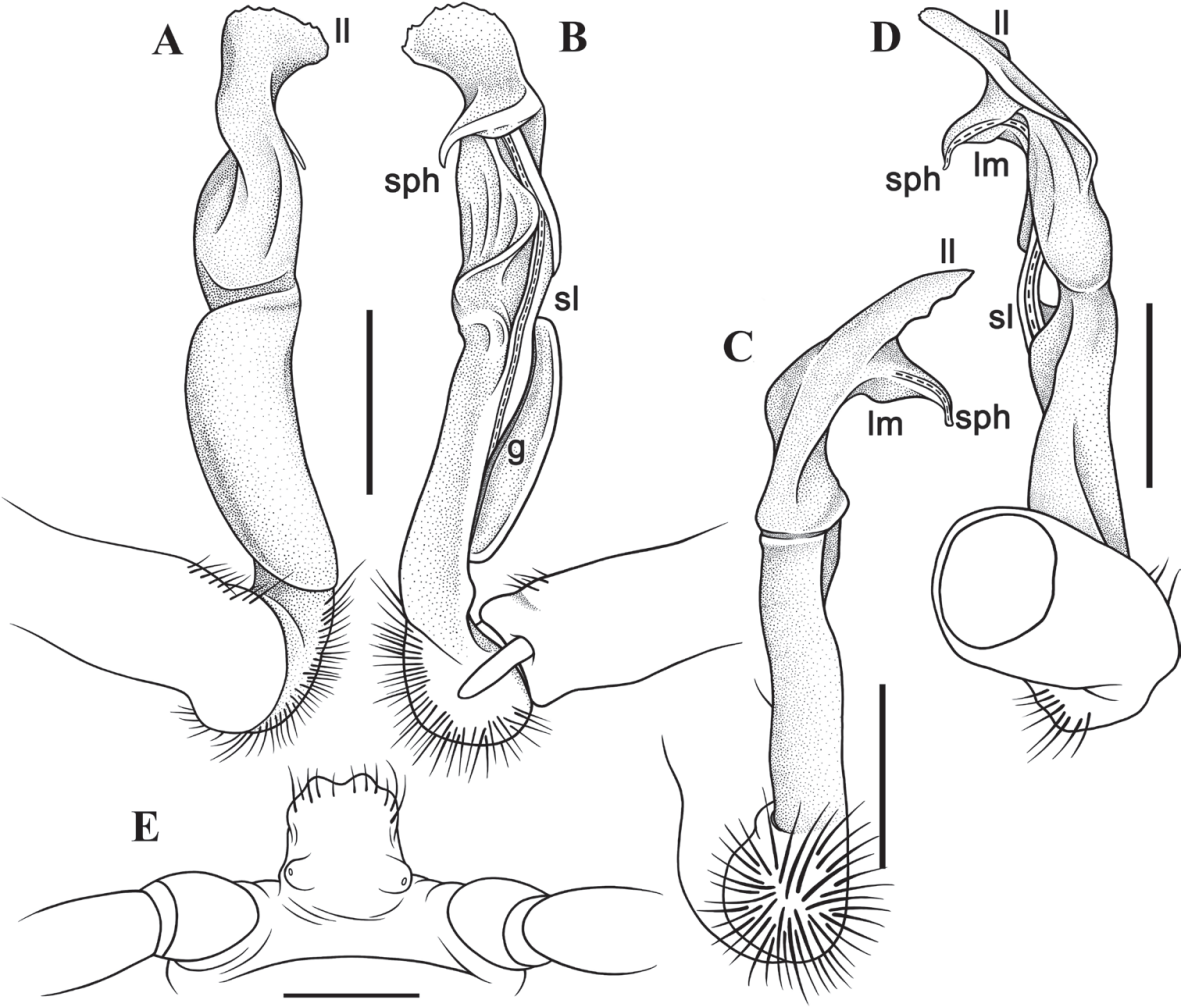
*Malayorthomorphahalabala* sp. nov., ♂ holotype, right gonopod **A–D** lateral, mesal, suboral and subcaudal views, respectively **E** sternal cones between coxae 4, subcaudal view. Abbreviations: **ll** lamina lateralis, **lm** lamina medialis, **sl** solenomere, **sph** = solenophore. Scale bars: 0.5 mm.

**Figure 5.**
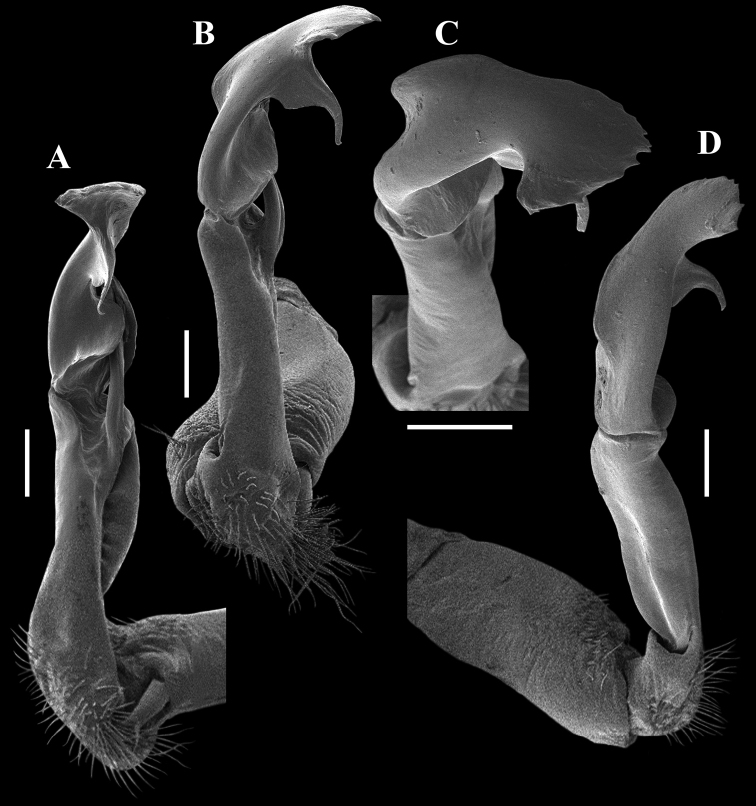
*Malayorthomorphahalabala* sp. nov., ♂ holotype, right gonopod **A–D** submesal, oral, subsuperior and sublateral views, respectively. Scale bars: 0.2 mm.

Gonopods (Figs 4A–D, 5) simple; coxa a little curved caudad, densely setose distoventrally. Prefemur as usual, densely setose, about 1/3 as long as femorite + postfemoral part. Femorite rather stout, wider than prefemur or postfemur, slightly expanded distad, suberect, showing a distinct mesal groove/hollow (**g**), with a sulcus demarcating a postfemoral part; seminal groove running entirely mesally along fermorite, solenomere (**sl**) flagelliform, almost fully sheathed by solenophore (**sph**). Lamina medialis (**lm**) well developed, short and unciform, terminal lobe sheathing the tip of solenomerite. Lamina lateralis (**ll**) elevated, prominent, stout, expanded apically, denticulate at caudal edge (Figs 3A, B, 4C, D).

##### Etymology.

To emphasize Hala-Bala Wildlife Sanctuary, the type locality. Noun in apposition.

##### Remarks.

A comparison of these two species shows only a few differences, but they are sufficient to distinguish both. The type locality of *M.siveci*, Park Belum, is located quite far away (ca. 50 km) from this new place. In addition, because the elevations between the two localities are greater than 1000 meters above sea-level, it seems improbable that the species is one and the same. Consequently, we conclude that the two are obviously distinct species.

The specimens were collected in a primary sub-elfin montane forest with no significant disturbance due to human activity, in a high mountainous area of southernmost Thailand (Fig. 2B, C). The area is dominated by a single plant species, *Dacrydiumelatum*. The canopy of *Dacrydiumelatum* is low (ca. 10–15 m above ground), flat and continuously covering the area. The understorey is dense and covered with dwarf branches of small hardwood trees and teeming with bryophytes, lichens, orchids and ferns. The forest floor is with abundant orchids, ferns, liverworts, and thick slowly degraded bio-litters. The female specimen was easy to spot on the substrate and observed crawling on the leaf litter surface (Fig. 1A).

#### ﻿ Malayorthomorpha hulutbeeda sp. nov.

Taxon classificationAnimaliaPolydesmidaParadoxosomatidae

4E5486C3-1E6D-5230-A5A3-10AB0C51AEF0

https://zoobank.org/49D76123-EB7C-4682-BD8F-48881036EDDC

Figs 6 , 7 , 8

##### Material examined.

***Holotype***: Thailand – Yala Province • ♂; Betong District, elfin montane forest (Malaya Phytochorion province); 1430 m a.s.l.; 25 May 2022; Wisut Sittichaya leg.; CUMZ.

##### Diagnosis.

This new species is distinguished from its two congeners in sternal process between male coxae 4 linguiform with a rounded tip, and lamina lateralis of gonopodal solenophore triangular, apically bifid and protruded laterally.

##### Description.

Length of holotype 31.5 mm, width of midbody pro- and metazonae 2.7 and 3.0 mm, respectively.

**Figure 6.**
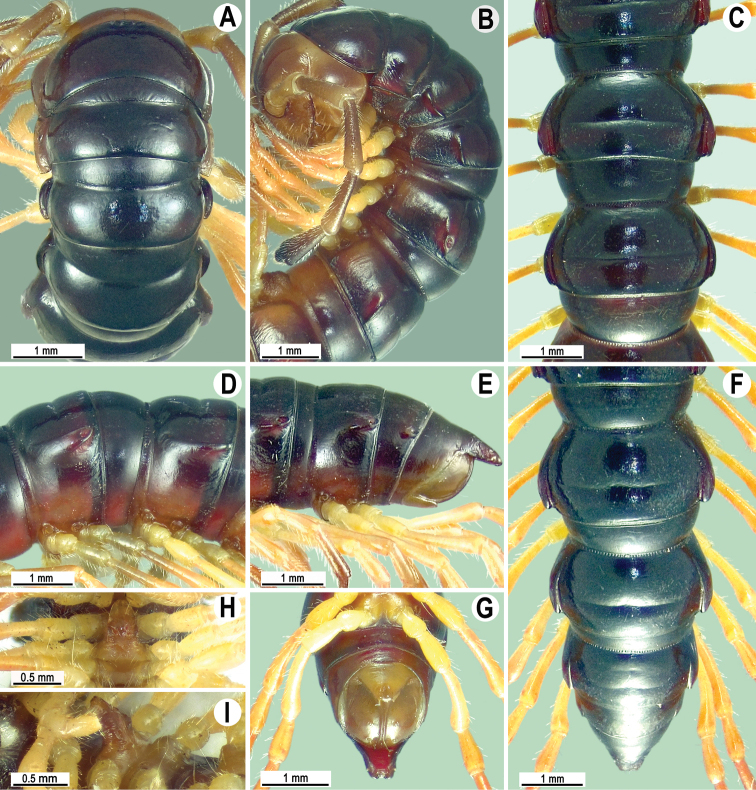
*Malayorthomorphahulutbeeda* sp. nov., ♂ holotype **A, B** anterior part of body, dorsal and lateral views, respectively **C, D** segments 10 and 11, dorsal and lateral views, respectively **E–G** posterior part of body, lateral, dorsal and subventral views, respectively **H, I** sternal cones between coxae 4, subcaudal and sublateral views, respectively

Colouration of alcohol material after one week of preservation dark red brown (Fig. 6A–F); paraterga paler, head and antennae light brown to brown (Fig. 6A, B), legs and venter contrasting light yellow to brown (Fig. 6), antennae and legs increasingly darker brown distally (Fig. 6B, E, G).

All characters as in *M.halabala* sp. nov., except as follows.

Antennae rather long, extending caudally past metaterga 4 when stretched dorsally. Collum with three transverse rows of setae: 4+4 in anterior, 2+2 in intermediate, and 3+3 in posterior row; with a small lateral setigerous incision near midway (Fig. 6A, B).

Paraterga 2 broad, anterior edge angular and rounded, lateral edge with a small notch at about 1/4 in front of caudal corner (Fig. 6A). Calluses on paraterga rather narrow, delimited by a sulcus fully on dorsal side and in posterior half on ventral side; on poreless rings narrower than on pore-bearing ones in dorsal view (Fig. 6B, D, E).

Transverse sulcus distinct (Fig. 6A, C, F), complete on metaterga 5–17, narrow, line-shaped, rather deep, not reaching the bases of paraterga, smooth at bottom, incomplete and nearly wanting on ring 18. Stricture between pro- and metazona wide, deep, beaded at bottom down to base of paraterga starting with segment 5 (Fig. 6A–F). Pleurosternal carinae complete crests with a sharp caudal tooth on rings 2–4, increasingly reduced and retaining a sharp caudal tooth on rings 5 and 6 thereafter, retaining a small caudal tooth on ring 7, missing further on (Fig. 6B, D, E).

Hypoproct roundly subtriangular, setigerous knobs at caudal edge very small and well-separated (Fig. 6G).

**Figure 7.**
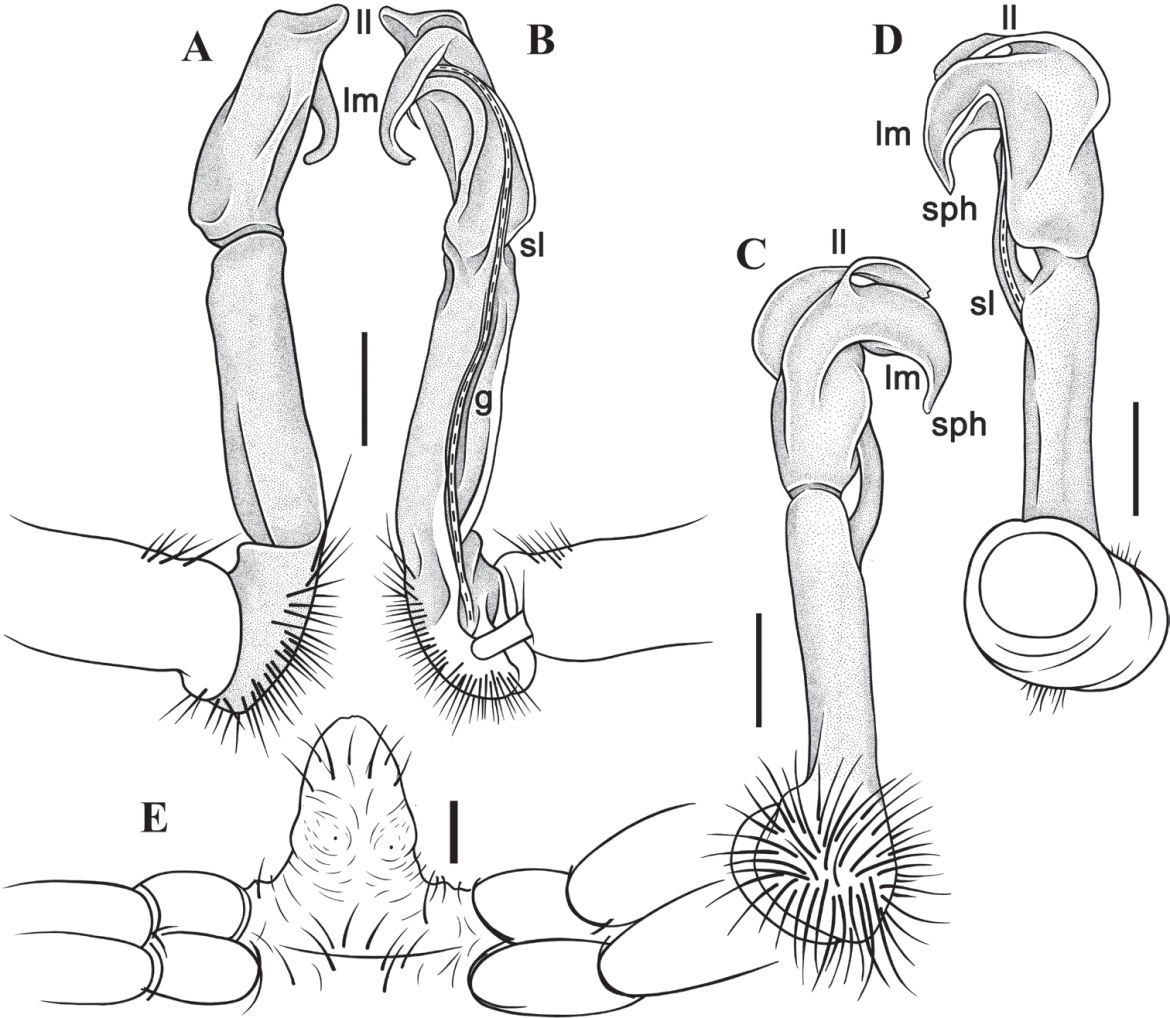
*Malayorthomorphahulutbeeda* sp. nov., ♂ holotype, right gonopod **A–D** lateral, mesal, oral and caudal views, respectively **E** sternal cones between coxae 4, subcaudal view. Abbreviations: **ll** lamina lateralis, **lm** lamina medialis, **sl** solenomere, **sph** solenophore. Scale bars: 0.2 mm.

Sterna moderately setose, shining, cross-impressions shallow, without modifications; an entire, large, linguiform, sternal lobe between ♂ coxae 4, with a pair of small denticles laterally near base (Figs 6H, I, 7E). An inconspicuous and low ridge present in front of gonopod aperture. Legs long and slender, midbody ones ca. 1.6–1.9 times as long as body height, without modifications, ♂ tarsal brushes absent.

**Figure 8.**
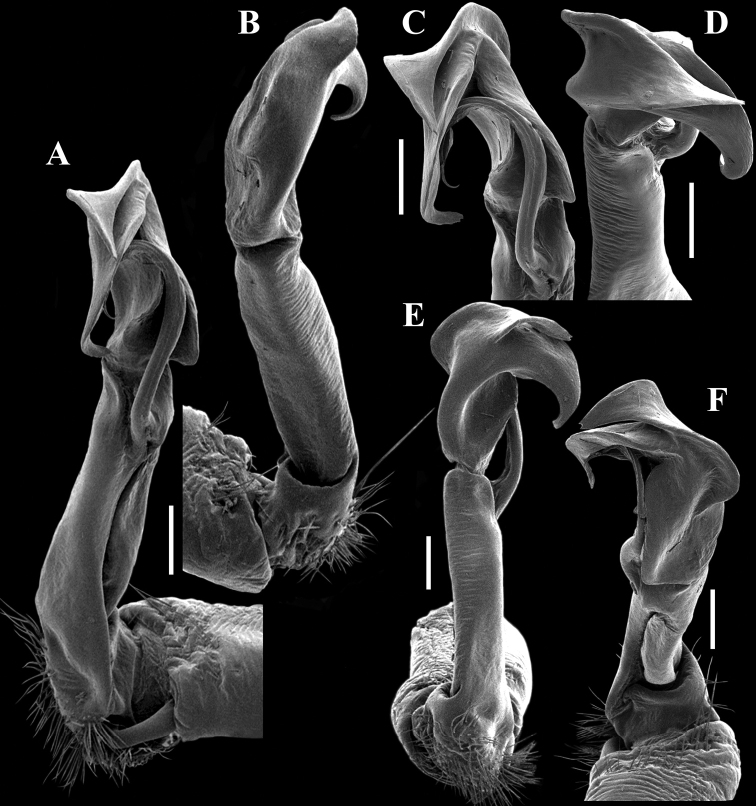
*Malayorthomorphahulutbeeda* sp. nov., ♂ holotype, right gonopod **A, B** mesal and lateral views, respectively **C–F** submesal, subsuperior, oral and subcaudal views, respectively. Scale bars: 0.2 mm

Gonopods (Figs 7A–D, 8) rather simple; coxa almost straight caudad, densely setose distoventrally. Prefemur as usual, densely setose, about 1/3 as long as femorite + postfemoral part. Femorite stout, suberect, showing a distinct mesal groove/hollow (**g**), with a sulcus demarcating a postfemoral part; seminal groove running entirely mesally along fermorite, solenomere (**sl**) flagelliform, almost fully sheathed by solenophore (**sph**). Lamina medialis (**lm**) well developed, thick and large, unciform, terminal lobe sheathing the tip of solenomere. Lamina lateralis (**ll**) triangular in shape, protruding laterally, tapered apically, bifid at tip (Figs 7C, D, 8A, 8C–D).

##### Etymology.

To emphasize “*hulutbeeda*” which means “flat-back millipede” in Malay dialect, a noun in apposition. A Malay dialect language is mainly used in three provinces of southern Thailand where the holotype was obtained.

##### Remark.

This species was found living together with *M.halabala* sp. nov. Moreover, according to our observations, they may even occur syntopically, sharing the same habitat: leaf litter surface, branches of trees and tree trunks.

## ﻿Discussion and conclusion

In accordance with the previous observations of related genera such as *Orthomorpha* Bollman, 1893, *Desmoxytes* Chamberlin, 1923 and *Tylopus* Jeekel, 1968, the coexistence of congeners is quite common to come across in Paradoxosomatidae generally and Orthomorphini in particular. So the syntopy of *Malayorthomorphahalabala* sp. nov. and *M.hulutbeeda* sp. nov. is not unusual. For example, *Desmoxytesplanata* (Pocock, 1895) was discovered beside *D.octoconigera* Srisonchai, Enghoff & Panha, 2018, *D.golovatchi* Srisonchai, Enghoff & Panha, 2018 and *D.purpurosea* Enghoff, Sutcharit & Panha, 2007 in several places (Srisonchai et al. 2018a). *Nagaxyteserecta* Srisonchai, Enghoff & Panha, 2018 and *N.gracilis* Srisonchai, Enghoff & Panha, 2018 were found jointly at Daowadueng Cave and Wat Sunantha Wanaram in Kanchanaburi Province, Thailand. Both latter species also show a very similar pattern of gonopodal structure (Srisonchai et al. 2018b). In the genus *Tylopus*, numerous species have been reported co-occurring in larger mountainous regions, such as Doi Inthanon (10 species) and Doi Suthep (10 species) in Thailand. However, some of them, at least in adult stages, appear to reflect separate phenofaunas that are restricted to relatively limited time periods and therefore do not overlap with others (Likhitrakarn et al. 2010, 2014, 2016). Therefore, it is far from surprising that both new species have been found to coexist at the same place. Although they share the same habitat, they may have distinct microhabitats, although this remains speculation at this stage.

*Malayorthomorpha* species are presently endemic to southern Thailand and northern Peninsular Malaysia, both of which are located within the Titiwangsa Mountain Range, which is known as Peninsular Malaysia’s backbone and longest mountain ridge. It begins in the north of southern Thailand, crosses the Malaysian border, enters the Negeri Sembilan valley, and terminates in the south near Jelebu, Negeri Sembilan (Chan et al. 2019). Mount Korbun is the highest peak in the Titiwangsa Range, reaching 2183 m above sea-level, and the second highest mountain in Peninsular Malaysia. In terms of biodiversity, the Mount Korbun area alone supports at least 18 amphibian, 134 bird, 42 mammal, and 18 reptile species, in addition to around 460 kinds of flowering plants and approximately 100 species of ferns and fern allies (Chan et al. 2019; Musthafa and Abdullah 2019). Due to the high biodiversity this mountain range supports, there are still many undiscovered species of flora and fauna. Thus, there are numerous unexplored millipede habitats in southern Thailand, particularly in the Pattani and Narathiwat provinces. Without doubt, new and exciting species will be discovered, and new localities reported, in this and surrounding regions in the future.

## Supplementary Material

XML Treatment for Malayorthomorpha

XML Treatment for Malayorthomorpha siveci

XML Treatment for Malayorthomorpha halabala

XML Treatment for Malayorthomorpha hulutbeeda

## References

[B1] AVMA (2013) AVMA guidelines for the euthanasia of animals. https://www.avma.org/KB/Policies/Documents/euthanasia.pdf [Accessed on: 2022-2-2]

[B2] Bhansali S Wesener T (2022) New Thai Giant Pill-Millipede species, with genetic barcoding data for Thai species (Diplopoda, Sphaerotheriida, Zephroniidae). Zootaxa 5105(3): 357–380. 10.11646/zootaxa.5105.3.2 35391297

[B3] Chan KO Muin MA Anuar S Andam J Razak N Aziz MA (2019) First checklist on the amphibians and reptiles of Mount Korbu, the second highest peak in Peninsular Malaysia. Check List 15(6): 1055–1069. 10.15560/15.6.1055

[B4] Demange JM (1961) Matériaux pour servir à une révision des Harpagophoridae. Mémoires du Muséum national d`Histoire naturelle, Sér. A (Zool.) 24: 1–274.

[B5] Enghoff H (2005) The millipedes of Thailand (Diplopoda). Steenstrupia 29(1): 87–103.

[B6] Golovatch SI (1993) On several new or poorly-known Oriental Paradoxosomatidae (DiplopodaPolydesmida). Arthropoda Selecta 2(1): 3–14. 10.15298/arthsel.30.1.01

[B7] Golovatch SI (1997) On several new or poorly-known Oriental Paradoxosomatidae (DiplopodaPolydesmida), V. Arthropoda Selecta 5(3/4): 131–141. [for 1996]

[B8] Golovatch SI (1998) On several new or poorly-known Oriental Paradoxosomatidae (DiplopodaPolydesmida), VI. Arthropoda Selecta 6(3/4): 35–46.

[B9] Golovatch SI Enghoff H (1993) Review of the millipede genus *Tylopus*, with descriptions of new species from Thailand (Diplopoda, Polydesmida, Paradoxosomatidae). Steenstrupia 19(3): 85–125.

[B10] Huber BA Petcharad B Bumrungsri S (2015) Revision of the enigmatic Southeast Asian spider genus *Savarna* (Araneae, Pholcidae). European Journal of Taxonomy 160(160): 1–23. 10.5852/ejt.2015.160

[B11] Jeekel CAM (1968) On the classification and geographical distribution of the family Paradxosomatidae (Diplopoda, Polydesmida). Academisch Proefschrift, Rotterdam, 162 pp.

[B12] Likhitrakarn N Golovatch SI Prateepasen R Panha S (2010) Review of the genus *Tylopus* Jeekel, 1968, with descriptions of five new species from Thailand (Diplopoda, Polydesmida, Paradoxosomatidae). ZooKeys 72: 23–68. 10.3897/zookeys.72.744 PMC3083004 21594103

[B13] Likhitrakarn N Golovatch SI Panha S (2011) Revision of the Southeast Asian millipede genus *Orthomorpha* Bollman, 1893, with the proposal of a new genus (Diplopoda, Polydesmida, Paradoxosomatidae). ZooKeys 131: 1–161. 10.3897/zookeys.131.1921 PMC3208436 22140329

[B14] Likhitrakarn N Golovatch SI Panha S (2014) Three new species of the millipede genus *Tylopus* Jeekel, 1968 from Thailand, with additional notes on species described by Attems (Diplopoda, Polydesmida, Paradoxosomatidae). ZooKeys 435: 63–91. 10.3897/zookeys.435.8086 PMC4141187 25152687

[B15] Likhitrakarn N Golovatch SI Panha S (2016) The millipede genus *Tylopus* Jeekel, 1968 (Diplopoda, Polydesmida, Paradoxosomatidae), with a key and descriptions of eight new species from Indochina. European Journal of Taxonomy 195(195): 1–47. 10.5852/ejt.2016.195

[B16] Likhitrakarn N Golovatch SI Jeratthitikul E Srisonchai R Sutcharit C Panha S (2020) A remarkable new species of the millipede genus *Trachyjulus* Peters, 1864 (Diplopoda, Spirostreptida, Cambalopsidae) from Thailand, based both on morphological and molecular evidence. ZooKeys 925: 55–72. 10.3897/zookeys.925.49953 32317853 PMC7160207

[B17] Likhitrakarn N Golovatch SI Jantarit S (2021a) Two new species of the millipede genus *Glyphiulus* Gervais, 1847 (Diplopoda, Spirostreptida, Cambalopsidae) from caves in northern Thailand. ZooKeys 1056: 173–189. 10.3897/zookeys.1056.71395 34522154 PMC8397691

[B18] Likhitrakarn N Golovatch SI Srisonchai R Sutcharit C (2021b) Two New Species of the Giant Pill-Millipede Genus *Zephronia* Gray, 1832 from Thailand (Diplopoda: Sphaerotheriida: Zephroniidae). Tropical Natural History 21(1): 12–26.

[B19] Likhitrakarn N Golovatch SI Panha S (2022) The Oriental millipede genus *Nepalella* Shear, 1979, with the description of a new species from Thailand and an updated key (Diplopoda, Chordeumatida, Megalotylidae). ZooKeys 1084: 183–199. 10.3897/zookeys.1084.78744 35233169 PMC8825426

[B20] Mršić N (1996) On three new Paradoxosomatidae from Malaysia (DiplopodaPolydesmida). Arthropoda Selecta 5(1/2): 139–144.

[B21] Musthafa MM Abdullah F (2019) Coleoptera of Genting Highland, Malaysia: Species richness and diversity changes along the elevations. Museu de Ciències Naturals de Barcelona. Miscel·lània. Zoològica 17: 123–144. 10.15470/i0uuis

[B22] Myers N Mittermeier RA Mittermeier CG Da Fonseca G Kent J (2000) Biodiversity hotspots for conservation priorities. Nature 403(6772): 853–858. 10.1038/35002501 10706275

[B23] Nguyen AD Sierwald P (2013) A worldwide catalog of the family Paradoxosomatidae Daday, 1889 (Diplopoda: Polydesmida). Check List 9(6): 1132–1353. 10.15560/9.6.1132

[B24] Pimvichai P Enghoff H Panha S (2009) A revision of the *Thyropygusallevatus* group. Part 1: the *T.opinatus* subgroup (Diplopoda: Spirostreptida: Harpagophoridae). Zootaxa 2016(1): 17–50. 10.11646/zootaxa.2016.1.2

[B25] Pimvichai P Enghoff H Panha S Backeljau T (2018) Morphological and mitochondrial DNA data reshuffle the taxonomy of the genera *Atopochetus* Attems, *Litostrophus* Chamberlin and *Tonkinbolus* Verhoeff (Diplopoda: Spirobolida: Pachybolidae), with descriptions of nine new species. Invertebrate Systematics 32(1): 159–195. 10.1071/IS17052

[B26] Pimvichai P Enghoff H Panha S Backeljau T (2020) Integrative taxonomy of the new millipede genus *Coxobolellus*, gen. nov. (Diplopoda: Spirobolida: Pseudospirobolellidae), with descriptions of ten new species. Invertebrate Systematics 34: 591–617. 10.1071/IS20031

[B27] Pocock RI (1889) Contributions to the fauna of Mergui and its archipelago, Volume 1: Myriopoda. Report on the Myriopoda of the Mergui Archipelago, collected for the Trustees of the Indian Museum, Calcutta, by Dr. John Anderson, F.R.S., Superintendant of the Museum. Zoological Journal of the Linnean Society 21: 287–330. 10.1111/j.1096-3642.1889.tb00980.x

[B28] Rosenmejer T Enghoff H Moritz L Wesener T (2021) Integrative description of new giant pill-millipedes from southern Thailand (Diplopoda, Sphaerotheriida, Zephroniidae). European Journal of Taxonomy 762: 108–132. 10.5852/ejt.2021.762.1457

[B29] Shelley RM Sierwald P Kiser SB Golovatch SI (2000) Nomenclator generum et familiarum Diplopodorum II. A List of the Genus and Family-Group Names in the Class Diplopoda from 1958 through 1999. Pensoft, Sofia, 167 pp.

[B30] Sinclair FG (1901) On the myriapods collected during the “Skeat Expedition” to the Malay Peninsula, 1899–1900. Proceedings of the Zoological Society of London 71(2): 505–533. 10.1111/j.1469-7998.1902.tb08186.x

[B31] Srisonchai R Enghoff H Likhitrakarn N Panha S (2018a) A revision of dragon millipedes I: Genus *Desmoxytes* Chamberlin, 1923, with the description of eight new species (Diplopoda, Polydesmida, Paradoxosomatidae). ZooKeys 761: 1–177. 10.3897/zookeys.761.24214 PMC5988806 29875597

[B32] Srisonchai R Enghoff H Likhitrakarn N Panha S (2018b) A revision of dragon millipedes II: The new genus *Nagaxytes* gen. nov., with the description of three new species (Diplopoda, Polydesmida, Paradoxosomatidae). European Journal of Taxonomy 462(462): 1–44. 10.5852/ejt.2018.462

[B33] Srisonchai R Enghoff H Likhitrakarn N Panha S (2018c) A revision of dragon millipedes III: The new genus *Gigaxytes* gen. nov., with the description of three new species (Diplopoda, Polydesmida, Paradoxosomatidae). European Journal of Taxonomy 463(463): 1–43. 10.5852/ejt.2018.463

[B34] Srisonchai R Enghoff H Likhitrakarn N Panha S (2018d) A revision of dragon millipedes IV: The new genus *Spinaxytes*, with the description of nine new species (Diplopoda, Polydesmida, Paradoxosomatidae). ZooKeys 797: 19–69. 10.3897/zookeys.797.29510 PMC6255853 30505161

[B35] Srisonchai R Likhitrakarn N Sutcharit C Jeratthitikul E Siriwut W Thrach P Chhuoy S Ngor PB Panha S (2020) A new micropolydesmoid millipede of the genus *Eutrichodesmus* Silvestri, 1910 from Cambodia, with a key to species in mainland Southeast Asia (Diplopoda, Polydesmida, Haplodesmidae). ZooKeys 996: 59–91. 10.3897/zookeys.996.57411 33312046 PMC7710679

[B36] Srisonchai R Sutcharit C Likhitrakarn N (2021) The giant pill-millipede genus *Zephronia* Gray, 1832 from Thailand, with a redescription of *Z.siamensis* Hirst, 1907 and descriptions of three new species (Diplopoda, Sphaerotheriida, Zephroniidae). ZooKeys 1067: 19–56. 10.3897/zookeys.1067.72369 34759718 PMC8571248

[B37] Wang YM Tang MC (1965) Seria 1R: The millipedes of Malay Archipelago and South Sea Islands: Singapore, Sarawak and Sumatra. Quarterly Journal of the Taiwan Museum 18: 399–441.

